# Gestational and Lactational Exposure to an Environmentally‐Relevant Mixture of Brominated Flame Retardants: Effects on Neurodevelopment and Metabolism

**DOI:** 10.1002/bdr2.1021

**Published:** 2017-03-24

**Authors:** Emily W. Y. Tung, Alice Kawata, Marc Rigden, Wayne J. Bowers, Don Caldwell, Alison C. Holloway, Bernard Robaire, Barbara F. Hales, Michael G. Wade

**Affiliations:** ^1^ Environmental Health Science and Research BureauHealth CanadaOttawaOntarioCanada; ^2^ Health Products and Food BranchHealth CanadaOttawaOntarioCanada; ^3^ Department of Obstetrics and GynecologyMcMaster UniversityHamiltonOntarioCanada; ^4^ Department of Pharmacology and TherapeuticsMcGill UniversityMontréalQuebecCanada; ^5^ Department of Obstetrics and GynecologyMcGill UniversityMontréalQuebecCanada

**Keywords:** brominated flame retardants, glucose tolerance, hexabromocyclododecane, neurodevelopment, polybrominated diphenyl ethers

## Abstract

**Background:**

Developmental exposure to brominated flame retardants (BFRs), including polybrominated diphenyl ethers (PBDEs) and hexabromocyclododecane (HBCDD), has been associated with impaired neurodevelopment and some symptoms of metabolic syndrome. However, there are inconsistencies in studies reporting neurodevelopmental effects with studies of pure substances more likely to report effects than studies of technical products. In addition, the influence of early BFR exposures on later development of metabolic disease‐like symptoms has not been investigated. This study examined the effects of perinatal exposure to an environmentally relevant mixture of BFRs based on relative levels observed in house dust, on several markers of neurodevelopment and metabolism in offspring.

**Methods:**

Sprague–Dawley female rats were fed a diet estimated to deliver daily doses of 0, 0.06, 20, or 60 mg/kg of a mixture of PBDEs and HBCDD from before mating to weaning. Offspring were weaned to control diet and subjected to neurobehavioral and metabolic assessments.

**Results:**

Exposure to BFRs decreased vertical movement in at postnatal day (PND) 32 and increased time to emerge to a lighted area on PND 105 in offspring of both sexes. Although early life exposure to the BFR mixture did not impact measures of glucose or insulin action, male offspring had significantly decreased fat pad weights at PND 46. Total cholesterol was increased in male and female offspring exposed to the highest dose at PND 21.

**Conclusions:**

These results suggest that gestational and lactational exposure to an environmentally relevant BFR mixture may induce changes in neurodevelopment and lipid metabolism in offspring. Birth Defects Research 109:497–512, 2017.© 2017 The Authors Birth Defects Research Published by Wiley Periodicals, Inc.

## Introduction

Brominated flame retardants (BFRs) are found in many commercial products, including furniture foam, electronics, foam insulation, and carpet padding (Alaee et al., 2003; Birnbaum and Staskal, 2004; Chen et al., 2014). As the majority of these are not covalently linked to the polymer matrices in which they are used, these substances can and do leach into indoor environments. They also tend to accumulate in human tissues because of their lipophilicity and resistance to biodegradation (Birnbaum and Staskal, 2004). The most widely used BFRs, the polybrominated diphenyl ethers (PBDEs), are of particular concern as these are routinely observed in every sample of house dust in North America (Allen et al., 2008; Dodson et al., 2012; Shoeib et al., 2012) and human exposures in utero are well documented (Doucet et al., 2009; Miller et al., 2012; Cowell et al., 2015). Early life exposures to these are estimated to be higher in toddlers and children compared with adults (Toms et al., 2009; Rawn et al., 2014) because of their ingestion of house dust from hand‐to‐mouth contact and of contaminated breast milk (Schecter et al., 2003; Jones–Otazo et al., 2005; Stapleton et al., 2008).

In animal models, exposure to PBDEs has been associated with a number of endocrine disrupting effects, including thyroid hormone antagonism and altered insulin signaling (Kojima et al., 2009; Suvorov et al., 2009). Our previous work has also shown that animals exposed to PBDEs or house dust‐based mixtures of BFR (mainly PBDEs with a small amount of hexabromocyclododecane [HBCDD]) during gestation and lactation show reduced serum levels of thyroid hormone (Berger et al., 2014; Bowers et al., 2015; Tung et al., 2016). As thyroid hormone is essential for normal brain development, PBDE‐induced alterations in thyroid hormone levels during critically vulnerable periods of brain development may result in permanent alterations of brain structure and function. There is accumulating evidence that early life exposures to BFRs are associated with neurodevelopmental disruptions based on toxicological and epidemiological studies (see Costa et al., 2014; Herbstman and Mall, 2014, for reviews). Several animal studies have reported changes in neurodevelopment after fetal or neonatal PBDE exposures. For example, perinatal exposure to low doses of the congener brominated diphenyl ether (BDE)‐47 increased spontaneous locomotor activity in exposed rat pups when compared with controls (Suvorov et al., 2009). In a separate study, BDE‐47 exposure caused decreased spatial memory in exposed mouse pups (Koenig et al., 2012). Moreover, gestational and lactational exposure to the congener BDE‐99 resulted in delayed negative geotaxic reflexes, in addition to impaired learning and memory (Cheng et al., 2009), whereas a second study revealed effects on spontaneous activity and reduced anxiety (Blanco et al., 2013). Although these studies and others have shown an association between developmental PBDE exposure and neurotoxicity, studies using the technical mixture DE‐71, a penta‐brominated mixture comprised of 36% BDE‐47 and 44% BDE‐99, revealed few alterations in neurobehavioral parameters after perinatal exposure, even though thyroid hormone levels were attenuated (Qiu et al., 2007; Kodavanti et al., 2010; Bowers et al., 2015). Although the reasons for such inconsistencies remain unknown, it has been suggested that both specific PBDE congeners and the timing of exposure may be critical factors (as summarized in Bowers et al., 2015). To date, there have been no studies to examine the neurodevelopmental effects in animal models of exposures to the full complement of PBDE congeners that would be experienced in the dust of built environments.

Furthermore, studies have shown a correlation between PBDE exposure and impaired human neurodevelopment. Recent birth cohort studies examining prenatal and childhood PBDE exposures reported a positive correlation between maternal PBDE serum concentrations during gestation and deficits in attention, fine motor coordination, and verbal comprehension in children at ages 5 to 7 years (Erkin–Cakmak et al., 2015), reduced scores on a variety of measures of motor function and IQ scores along with increased hyperactivity (Chen et al., 2014) and increased impulsive behaviors (Hoffman et al., 2012). These studies strongly suggest an association between PBDE exposure and alterations in learning and behavior in humans, yet the lack of consistent corroboration by animal data suggest either that the observed human effects are a consequence of a consistent confounder or that the animal models are not assessing the relevant exposures or neurological outcomes effectively.

In addition to effects on neurodevelopment, exposures to PBDEs may contribute to glucose intolerance, an increased risk of diabetes, or related metabolic disease. A human cross sectional study from the United States has reported that increasing serum levels of a PBDE congener (BDE‐153) were associated with an increased frequency of diabetes or clinical signs associated with metabolic disease (National Health and Nutrition Examination Survey) (Lim et al., 2008), whereas a case control study found that diabetes cases had significantly higher exposures to BDE‐47 (Zhang et al., 2016). Moreover, early life exposure to BDE‐47 in rats caused increased glucose uptake and plasma insulin‐like growth factor 1 levels in male offspring (Suvorov et al., 2009), whereas exposure to the technical mixture DE‐71 affected glucose/insulin ratios in rats (Nash et al., 2013). Exposure to DE‐71 also induced transcriptional markers of type 2 diabetes in the livers of adult rats (Dunnick et al., 2012). Evidence for effects on obesity is less clear. Although, PBDEs have been shown to induce fat cell differentiation in in vitro (Bastos Sales et al., 2013; Tung et al., 2016) exposures of humans to PBDEs during fetal or early postnatal life have been shown to have either no effect on body fat levels (Erkin–Cakmak et al., 2015) or was associated with reduced measures of obesity in children (Hoffman–Riz et al., 2016; Vuong et al., 2016). In animal studies, body weight was increased in both male and female offspring in the first few months of development after perinatal exposure to the congener BDE‐47 (Suvorov et al., 2009). Despite these suggestive observations, the potential for perinatal exposures to the full spectrum of PBDE congeners present in house dust to influence the development of glucose metabolism and adiposity in the exposed animal as an adult has not been investigated.

The current study builds on our previously published work that characterized developmental and toxicological outcomes after gestational and lactational exposure to the environmentally relevant mixture of BFRs (Tung et al., 2016). We reported that exposure to an environmentally relevant mixture of BFRs, based on levels observed in house dust, led to a reduction in serum T4 levels at postnatal day (PND) 21 in both male and female offspring, indicating the potential for neurodevelopmental anomalies. The current work reports neurodevelopment effects and impacts indicating metabolic syndrome in these offspring.

## Materials and Methods

### Chemicals

DE‐71 was a gift from Doug Arnold (Health Canada), from stock provided by Chemtura (Lawrenceville, GA). DE‐79 was purchased from Wellington Laboratories (Guelph, Ontario, Canada), and BDE‐209 (>97% pure) was purchased from Sigma–Aldrich (St. Louis, MO). The HBCDD mixture was a gift from Ivan Curran (Health Canada).

### Treatments

Diets containing an environmentally relevant mixture of BFRs, with relative levels of PBDE congeners and HBCDD based on median levels of these observed in North American house dust were fed to female Sprague–Dawley rats for a minimum of 1 week before mating, throughout gestation, and after delivery until weaning. The basis of this mixture, its formulation, and incorporation into diets has been described at length elsewhere (Ernest et al., 2012; Berger et al., 2014; Tung et al., 2016). Briefly, median levels of the PBDEs and HBCDDs observed in the Boston house dust studies (Allen et al., 2008; Stapleton et al., 2008) were used to determine the proportions of DE‐71, DE‐79, BDE‐209, and HBCDD that were subsequently incorporated into an isoflavone‐free diet (Teklad Global 2019 diet; Harlan Laboratories, Madison, WI) with 4.3 g/kg corn oil. Diets were prepared using three technical PBDE mixtures (DE‐71, DE‐79, and BDE‐209) and one HBCDD mixture to give 0 (control), 0.75, 250, or 750 mg of BFR mixture per kg of diet, with target nominal doses of 0, 0.06, 20, and 60 mg/kg of body weight per day based on a daily food consumption of 80 gm/kg body weight per day. Analyses of dietary content of specific congeners were reported previously (Berger et al., 2014).

### Animals

A full description of the animal breeding and litter treatment for this study has been published (Tung et al., 2016). Briefly, virgin female Sprague–Dawley rats (6–8 weeks old; Charles River, St‐Constant, Quebec, Canada) were acclimatized for 2 weeks on control diet. Animals were housed singly in hanging polycarbonate cages on hardwood chip bedding and provided with various enrichments (shelter, hardwood chew sticks, and marbles) and unlimited access to food and water (reverse osmosis‐treated municipal tap water). Animals were then randomized to treatment groups and provided with the diets containing 0, 0.75, 250, or 750 mg/kg of BFRs for at least 1 week before mating (*n* = 22–23/group). After this week‐long exposure period, female rats showing vaginal proestrus were housed overnight with proven male breeders. Mating was confirmed by a sperm‐positive vaginal swab and the day that this was observed was denoted as gestational day 0. All females were allowed to deliver (denoted as PND 0) and pups were counted with as little disturbance of the litter as possible. Dams were maintained on their respective diets throughout gestation and lactation to PND 21. Food consumption and dam and litter body weights were recorded at intervals throughout the treatment period. All animals were housed under controlled light (12 hrs of light:12 hrs of dark), humidity (40 to 70%), and temperature (20 to 24°C). All procedures were completed in accordance with the guidelines of the Canadian Council on Animal Care, and experimental procedures were approved by the Health Canada Animal Care Committee (Protocol ACC #2012‐015).

On PND 4, pups were recounted, the sexes of the pups were recorded, and the litter size was reduced to eight pups per litter (as close to four of each sex as possible). Animals were weaned at PND 21 on regular rodent chow (2019 Teklad Global) and littermates of the same sex and treatment were cohoused (2–4 per cage, depending on the sex ratio of the litter). One male and one female offspring per litter were randomly allocated to the neurobehavioral assessment cohort and transferred to a new cage to be cohoused with a pup of the same sex from a different litter within the same treatment group. Metabolic effects were assessed in a separate group of pups (one per sex per litter) and were subsequently necropsied at PND 210.

### Tissue Preparations

One male and one female offspring from each litter were euthanized at PNDs 21, 46, and 210 by exsanguination (abdominal aorta) under isoflurane anesthetic. Various tissues (liver, pancreas, and adrenal glands) as well as fat pads were dissected and weighed. Blood was transferred to a Vacutainer SST (BD Biosciences Canada, Mississauga, Ontario, Canada), allowed to clot for 30 min at room temperature, and kept on ice before centrifugation; serum aliquots were stored at –80°C before further analyses.

### Measurement of Serum Cholesterol

Total cholesterol, high‐density lipoprotein (HDL), low‐density lipoprotein (LDL), and triglycerides were measured using an ABX Pentra 400 clinical chemistry analyzer (Horiba ABX, Montpellier, France).

### Neurobehavioral Assessments

One male and one female per litter were assigned for neurobehavioral testing. The following tests were administered: motor activity (at PND 35 for a 30‐min block; 85, and 140 PND for 60‐min blocks), startle response (PND 40), and emergence latency (PND 42 and PND 100). All testing occurred during the light cycle.

For motor activity, testing was done in a chamber (Model ENV‐515; Med Associates, St. Albans, VT) with a lower set of arrays (16 × 16 beams) detecting lateral movement and a higher set of identical arrays detecting vertical movement (rearing behavior). Motor activity measures included ambulatory counts, ambulatory distance, ambulatory time, vertical counts, vertical time, and resting time.

Startle testing was conducted using San Diego Instruments Startle Reflex Laboratory Equipment running SR‐Lab Startle Reflex System software (release 6500‐0091, version 5.0). Animals were placed in an acrylic holder on a transducer platform inside a sound‐attenuated chamber. All platform potentiometers were normalized to animal weights before testing. All animals were acclimatized for 5 min in the chamber before the start of testing that consisted of 50 trials of 106 dbC broadband noise (30 msec duration) on a VI 20‐second schedule (10 to 20‐second range). Background noise level was 65 dbC and lighting inside the startle chamber was off for the duration of testing.

Testing for emergence latency was conducted in activity chambers (Model ENV‐515; Med Associates) divided into two equal sections by an opaque insert; one side was covered by a black opaque lid and the other illuminated by overhead lights. At the start of each session, subjects were placed in the dark‐side facing into the corner and clear Plexiglas lids were placed over each chamber. Animals were tested for a period of 10 min during which the times to emerge from the dark area and the time spent in the lighted and dark areas were recorded. The time of emergence from the dark area was defined as the time when the center of the animal first emerged at least 2.5 inches into the lighted area. Animals that did not emerge during the 10‐min test were assigned a latency value of 600 seconds. Details of test design and the modifications to ensure accurate testing are provided elsewhere (Bowers et al., 2015).

### Assessment of Glucose / Insulin Tolerance

A second cohort of animals consisting of one male and one female per litter was assessed for a variety of measures of glucose metabolism to determine if perinatal BFR exposures influenced glucose tolerance over the exposed animal's lifetime. These measures included fasted glucose and insulin to determine, and homeostasis model assessment‐insulin resistance (HOMA‐IR) at PND 25, PNDs 60 to 61, and PNDs 149 to 153, oral glucose tolerance test (OGTT), and an i.p. insulin tolerance test. For HOMA‐IR, the animals were fasted overnight and a single blood sample was collected from the tail vein into a Vacutainer SST tube (BD Biosciences Canada). Glucose levels were obtained from an additional small drop of whole blood using a handheld glucometer (NovaMax, Billerica, MA). Serum insulin levels were measured by ELISA (Crystal Chem, Downers Grove, IL). HOMA‐IR was calculated using the HOMA Calculator (https://www.dtu.ox.ac.uk/homacalculator).

OGTT were completed in one male and one female per litter at two timepoints: between PNDs 86 and 91 and between PNDs 177 and 183. As insulin secretion and action are highly influenced by ovarian steroid hormones, OGTT was assessed in female pups only during diestrus. Before testing, vaginal cytology was monitored daily by vaginal lavage to anticipate days of diestrus before overnight fasting. The cycle stage postfasting was confirmed and only those showing diestrus were tested. After an overnight fast, a blood sample (approximately 100 μl) was collected from the tail vein followed immediately by an oral bolus of glucose (2 gm/kg body weight) administered by gavage. Blood samples were drawn from the same site at 30, 60, and 120 min postgavage. Serum was prepared and stored at –80°C before insulin measurement by ELISA, whereas glucose was measured in whole blood at each timepoint, as described above. Insulin tolerance tests were conducted between PNDs 191 and 196. Only females in diestrus, assessed as described above, were tested. After a 6‐hr fast, glucose was collected and measured (as described above) and an i.p. injection of insulin (0.8 IU/kg) was administered. Blood glucose was further measured at 30, 60, and 120 min after injection.

### Histology

A sample of the median lobe of the liver was collected from the final necropsy (PNDs 206–210), fixed in 10% neutral‐buffered formalin, embedded in paraffin, sectioned at 10 μM, and stained with hematoxylin and eosin. One male and one female from five different litters in each treatment group were randomly selected for evaluation of hepatic morphological changes by an individual pathologist who was blinded to treatment. Lesions were subjectively assigned a numerical score from 0 to 5 indicating increasing severity of the lesion.

### Statistical Analyses

Data were analyzed using one‐way ANOVA followed by the Holm–Sidak post‐hoc test. When the assumption of normality or homogeneity of variance was not satisfied, data were analyzed by Kruskal–Wallis ANOVA on ranks (SigmaPlot version 12.0). For time courses in oral glucose tolerance and neurobehavioral tests, repeated‐measures ANOVA was used. Statistical significance was denoted when *p* < 0.05.

## Results

As previously reported (Tung et al., 2016), successful pregnancies were observed in 20, 11, and 17 females from the control group, and 0.75, 250, and 750 mg in the BFR diet group, respectively. Other results on litter outcomes, growth rates, and feed consumption, pup skeletal anomalies, and organ weights were also previously reported (Tung et al., 2016).

### Neurobehavioral Outcomes

**Figure 1:**
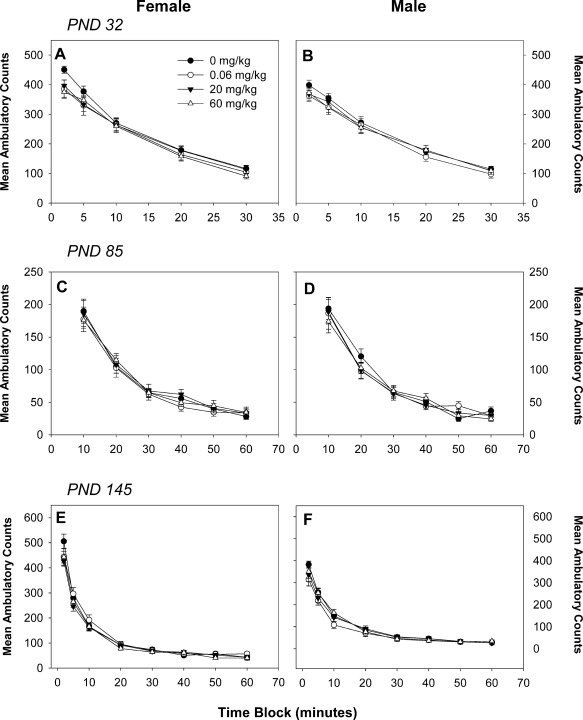
Motor activity as assessed by ambulatory counts in female (**A**, **C**, **E**) and male (**B**, **D**, **F**) offspring at postnatal day (PND) 32 (**A**, **B**), PND 90 (**C**, **D**), and PND 140 (**E**, **F**). Numbers 20, 10, and 15, with 15 for 0, 0.06, 20, and 60 mg/kg, respectively, at all timepoints. Values are expressed as mean ± SEM per treatment.

**Figure 2:**
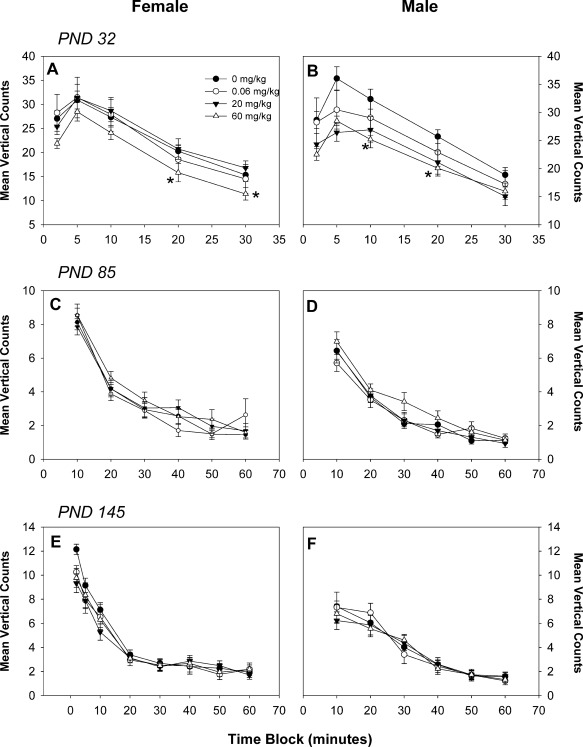
Motor activity as assessed by vertical counts in female (**A**, **C**, **E**) and male (**B**, **D**, **F**) offspring at postnatal day (PND) 32 (**A**, **B**), PND 90 (**C**, **D**), and PND 140 (**E**, **F**). Numbers 20, 10, and 15, with 15 for 0, 0.06, 20, and 60 mg/kg, respectively, at all time points. *p < 0.05 compared to the control group. Values are expressed as mean ± SEM per treatment.

Motor activity was assessed by ambulatory counts and distance, in addition to vertical movement. Ambulatory counts showed a normal habituation pattern for all ages; treatment‐related effects were not observed at PNDs 32, 85, or 145 in either male or female offspring for ambulatory counts (Fig. 1) or distance travelled (data not shown). Vertical movement, as assessed by vertical counts, was significantly decreased in PND 32 pups of either sex by exposure to 60 mg/kg BFR mixture (Fig. 2A, B for females and males, respectively); this effect was not observed at later ages (Fig. 2C–F).

**Figure 3:**
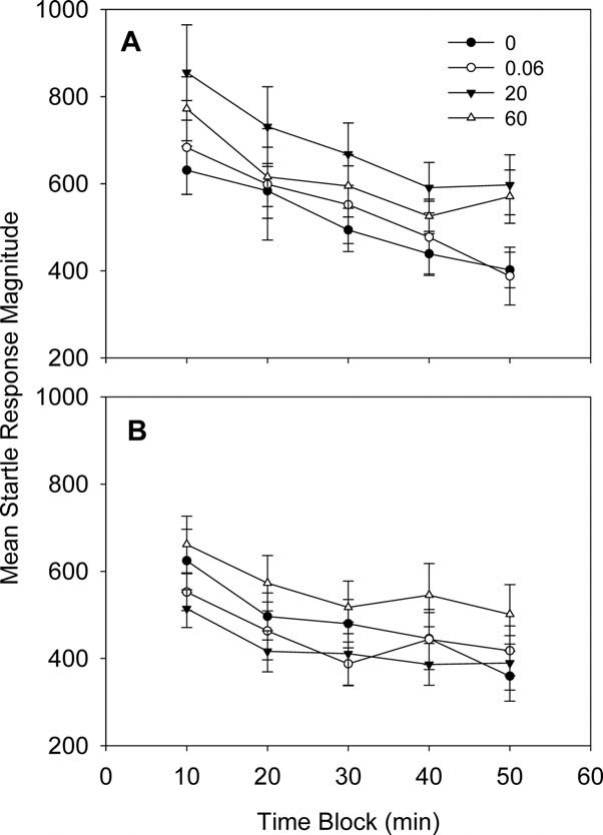
Startle response magnitude in female (**A**) and male (**B**) offspring at postnatal day (PND) 40. For males, numbers 15, 9, and 13, with 13 for control, 0.06, 20, and 60 mg/kg, respectively. For females, numbers 20, 10, and 15, with 15 for control, 0.06, 20, and 60 mg/kg, respectively. Values are expressed as mean ± SEM per treatment.

**Figure 4:**
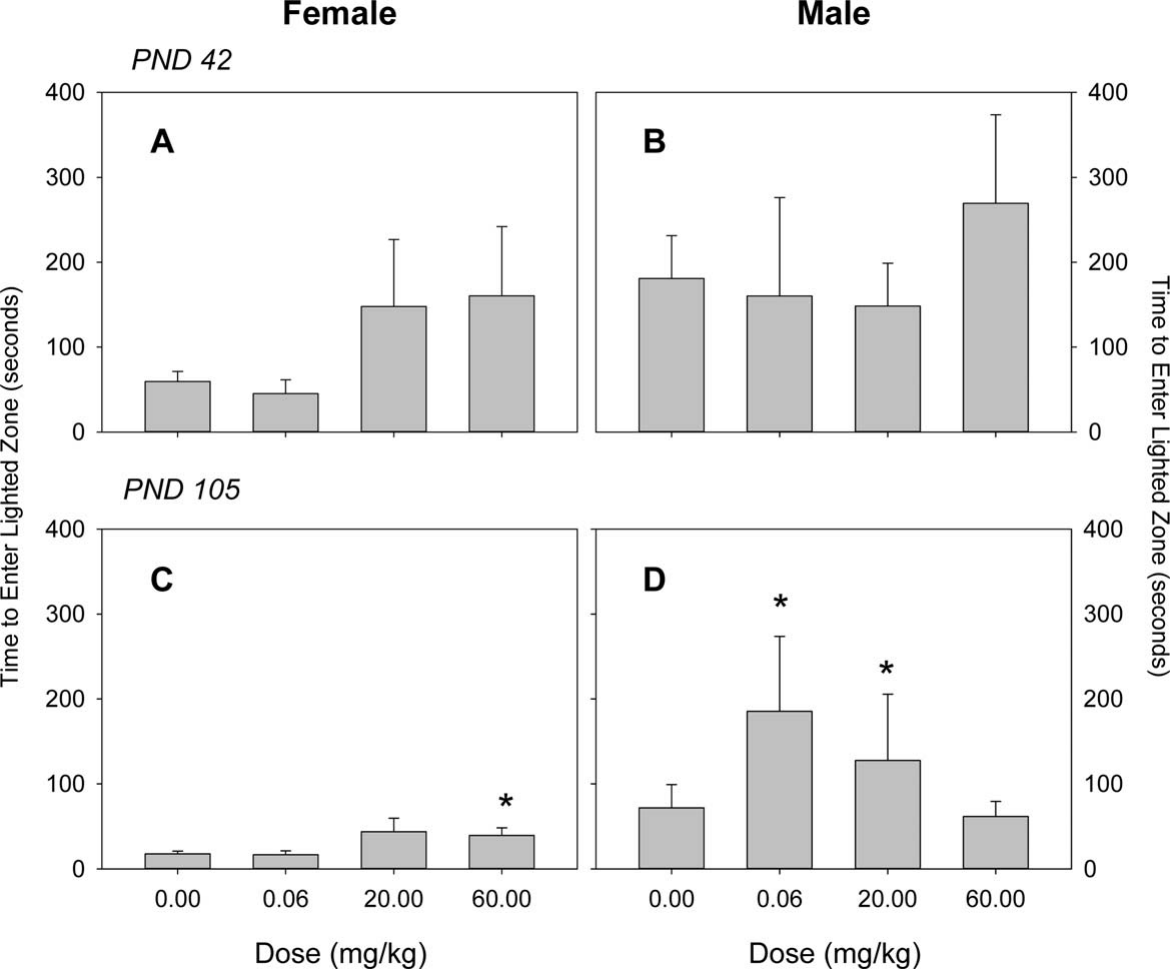
Emergence latency as assessed by time to enter lighted zone in female (**A**, **C**) and male (**B**, **D**) offspring at postnatal day (PND) 42 (**A**, **B**) and PND 100 (**C**, **D**). Values are expressed as mean ± SD per treatment. At PND 42, numbers 20, 10, and 15, with 15 for control, 0.06, 20, and 60 mg/kg, respectively, for both male and female offspring. At PND 105, numbers 20, 10, and 14, with 15 for control, 0.06, 20, and 60 mg/kg, respectively, for females. For male offspring at PND 105, numbers 20, 10, and 15, with 15 for control, 0.06, 20, and 60 mg/kg, respectively. *p < 0.05 compared to the control group. Values are expressed as mean ± SD per treatment.

Both male and female offspring exhibited normal startle habituation. BFR treatment did not influence the magnitude of startle response in male or female offspring at PND 40 (Fig. 3). Measurements of emergence latency showed no effect of treatment at PND 42 (Fig. 4A, B). At PND 105, however, male animals were consistently longer to emerge in all dose groups and were significantly further delayed in emergence by the 0.06 and 20 mg/kg BFR treatment, whereas exposure to 60 mg/kg BFR mixture did not influence the time to emerge (Fig. 4D). Emergence of female pups exposed to 20 and 60 mg/kg occurred, on average, threefold longer compared to the control group, but this effect reached statistical significance only in the highest dose at PND 105 (Fig. 4C).

### Measures of Metabolic Programming

Adipose tissue accumulation in pups was estimated by dissecting and weighing discrete fat depots. BFR treatment did not affect the weights of mesenteric, perirenal, epididymal or periovarian fat pads at PND 21 (Table 1). However, perirenal, epididymal, and testicular fat pads were all significantly reduced in weight (normalized to body weight) in male offspring at PND 46 after exposure to 20 mg/kg BFR mixture when compared to controls (*p* = 0.014, *p* = 0.008, and *p* < 0.001 for perirenal, epididymal, and testicular fat pads, respectively). No effects were observed in other fat pads for either male or female offspring at PND 46 in the other treatment groups. At PND 210, a significant reduction in relative periuterine fat pad weight was found in female offspring exposed to 20 mg/kg BFR mixture (*p* = 0.009).

**Table 1:** Fat pad and body weights.

	Dose, mg/kg							
	Male				Female			
	0	0.06	20	60	0	0.06	20	60
PND 21								
Number	19	10	17	16	20	10	17	15
Body weights	56.7 ± 4.0	55.8 ± 5.4	55.4 ± 4.3	54.8 ± 6.1	56.0 ± 4.2	54.7 ± 4.5	54.7 ± 4.6	53.9 ± 6.3
Mesenteric	0.56 ± 0.02	0.52 ± 0.002	0.49 ± 0.03	0.48 ± 0.03	0.57 ± 0.03	0.52 ± 0.03	0.49 ± 0.02	0.51 ± 0.02
Perirenal	0.24 ± 0.04	0.19 ± 0.04	0.22 ± 0.04	0.23 ± 0.03	0.26 ± 0.05	0.18 ± 0.04	0.23 ± 0.04	0.27 ± 0.04
Epididymal	0.12 ± 0.03	0.10 ± 0.04	0.11 ± 0.03	0.15 ± 0.03	–	–	–	–
Periovarian	–	–	–	–	0.14 ± 0.01	0.10 ± 0.01	0.12 ± 0.01	0.16 ± 0.02
PND 46								
Number	17	9	16	13	16	10	13	14
Body weights	230 ± 19	229 ± 15	217 ± 17	226 ± 25	176 ± 9	174 ± 13	166 ± 9	167 ± 13^a^
Mesenteric	0.58 ± 0.04	0.61 ± 0.04	0.50 ± 0.02	0.56 ± 0.02	0.62 ± 0.02	0.58 ± 0.06	0.63 ± 0.07	0.60 ± 0.03
Perirenal	1.02 ± 0.08	1.00 ± 0.07	0.75 ± 0.05^a^	0.92 ± 0.05	1.26 ± 0.07	1.34 ± 0.14	1.02 ± 0.09	1.26 ± 0.10
Epididymal	0.031 ± 0.0001	0.032 ± 0.003	0.025 ± 0.001^a^	0.027 ± 0.002	–	–	–	–
Testicular	1.14 ± 0.05	1.03 ± 0.06	0.86 ± 0.05^b^	0.99 ± 0.04	–	–	–	–
Periovarian	–	–	–	–	0.29 ± 0.01	0.32 ± 0.03	0.24 ± 0.01	0.27 ± 0.02
PND 210								
Number	19	10	17	15	20	11	17	16
Body weights	532 ± 43	516 ± 45	523 ± 37	524 ± 48	304 ± 63	286 ± 28	275 ± 23	283 ± 36
Mesenteric	0.96 ± 0.07	0.96 ± 0.14	0.80 ± 0.04	0.87 ± 0.08	0.69 ± 0.05	0.79 ± 0.05	0.64 ± 0.03	0.65 ± 0.05
Perirenal	3.25 ± 0.20	2.95 ± 0.22	2.92 ± 0.14	3.18 ± 0.26	2.43 ± 0.12	2.20 ± 0.16	2.01 ± 0.16	2.07 ± 0.16
Epididymal	0.11 ± 0.006	0.11 ± 0.01	0.11 ± 0.004	0.09 ± 0.006	–	–	–	–
Testicular	2.54 ± 0.11	2.45 ± 0.18	2.38 ± 0.12	2.44 ± 0.14	–	–	–	–
Periovarian	–	–	–	–	0.92 ± 0.06	0.93 ± 0.08	0.87 ± 0.04	0.81 ± 0.05
Periuterine	–	–	–	–	0.85 ± 0.07	0.71 ± 0.04	0.63 ± 0.04^a^	0.72 ± 0.05

Values are expressed as mean fat pad weight normalized to body weight ± SD.

a *p* < 0.05.

b *p* < 0.001.

PND, postnatal day.

**Figure 5:**
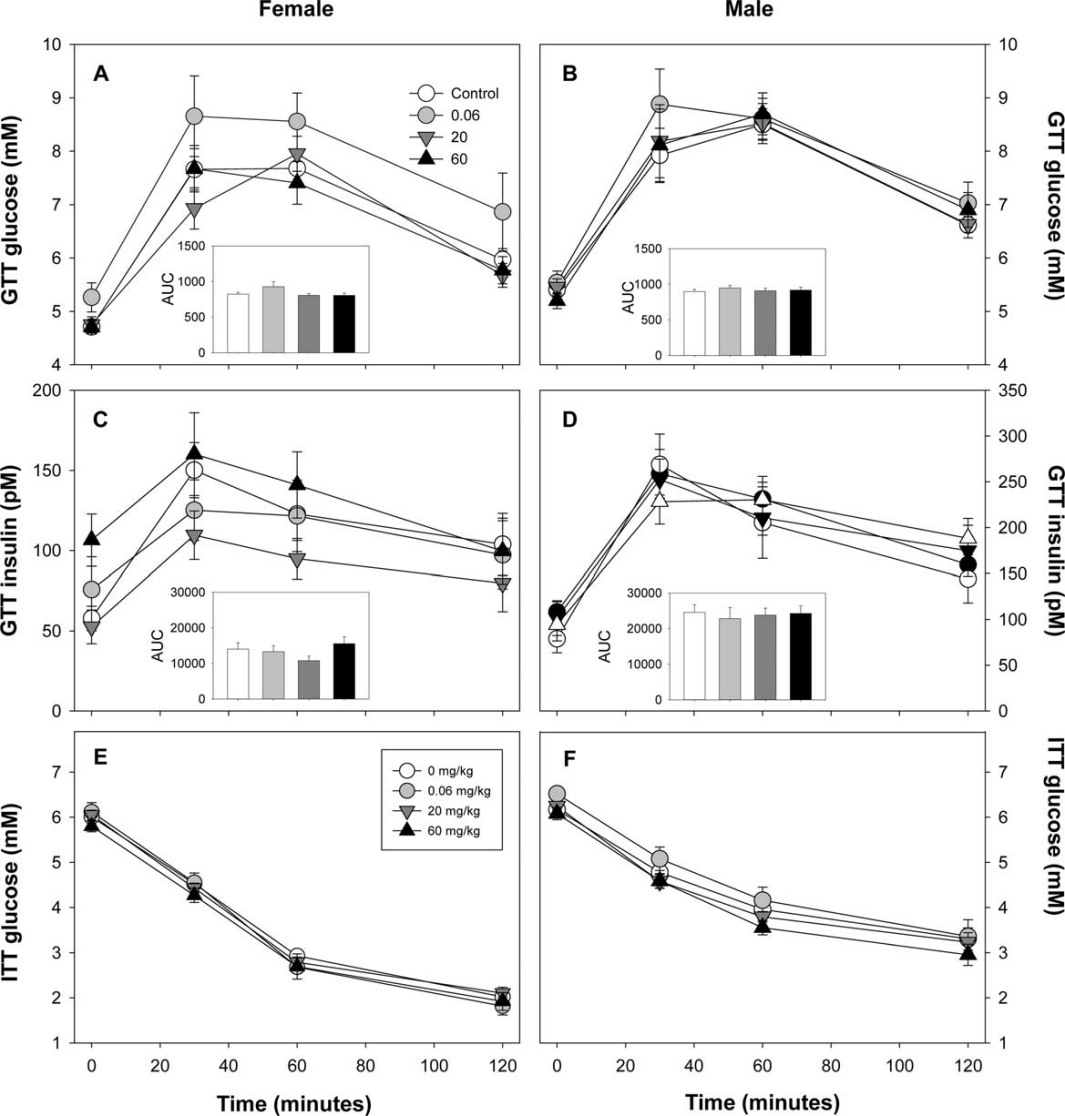
Blood glucose levels in female (**A**) and male (**B**) offspring after gavage of 2 gm/kg glucose. Total area under the curve (AUC) calculated for glucose for each is provided in the inset graph. Corresponding measures of serum insulin are shown for female (**C**) and male (**D**) pups. The calculated AUC for insulin is also depicted in the insets of each graph. For 0, 0.06, 20, and 60 mg/kg, numbers 18, 10, 17, and 15 for males and numbers 20, 11, 17, and 15 for females. Data expressed as mean ± SEM.

There was no effect of BFR treatment on fasting glucose, fasting insulin, HOMA‐IR, the glucose or insulin response to the glucose challenge, or insulin sensitivity in either male or female offspring (Fig. 5 and Suppl Tables S1–S5).

**Figure 6:**
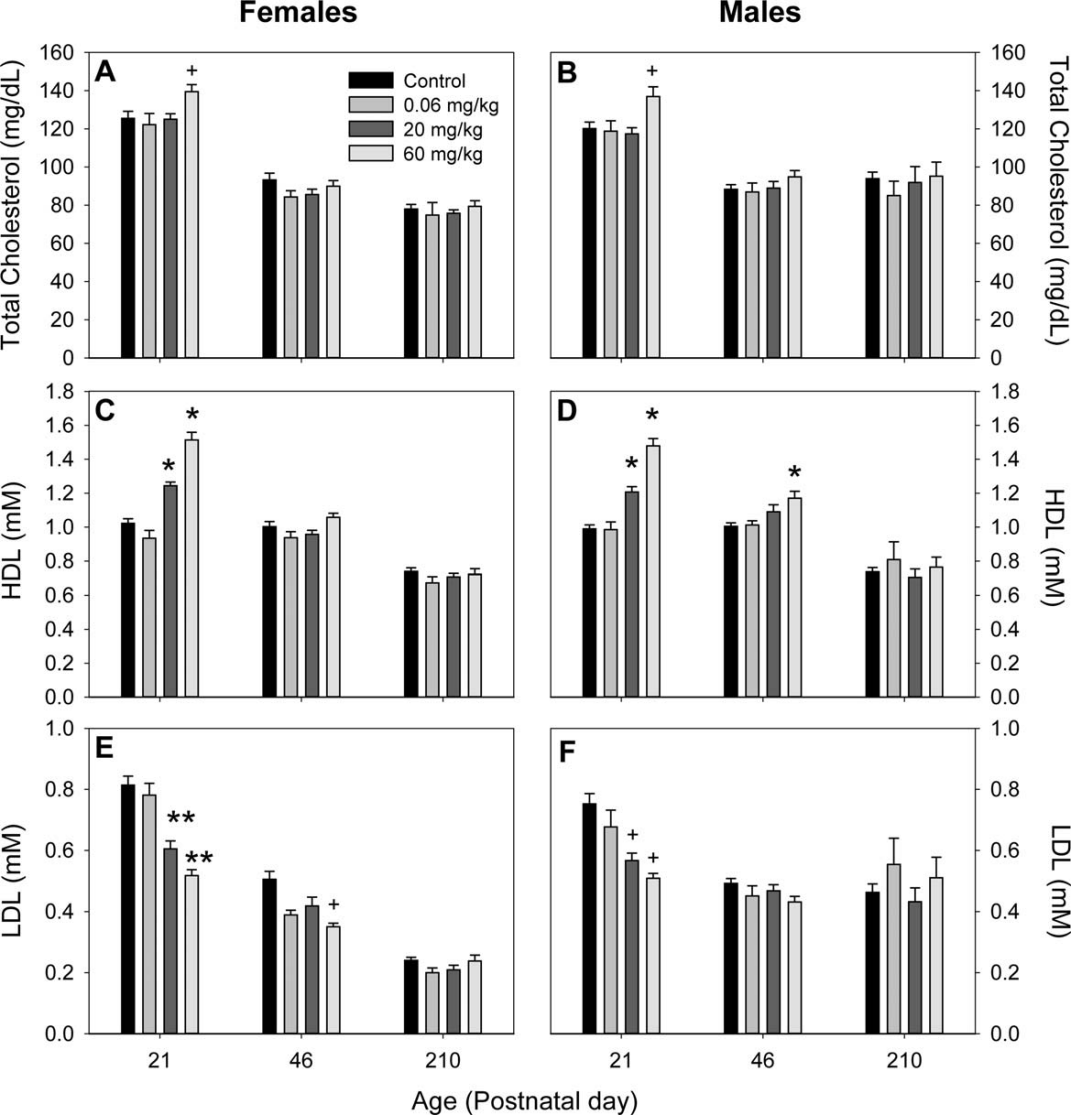
Serum levels of total cholesterol (**A**, **B**), high‐density lipoprotein (HDL) cholesterol (**C**, **D**), and low‐density lipoprotein (LDL) cholesterol measured in female (**A**, **C**, **E**) and male (**B**, **D**, **F**) pups at postnatal days (PNDs) 21, 46, and 210. +*p* < 0.05; *p < 0.01; **p < 0.001. Values expressed as mean ± SEM.

Serum measures of cholesterol and triglycerides were examined at PNDs 21, 46, and 210. At PND 21, total cholesterol levels in both male and female offspring were significantly elevated by exposure to 60 mg/kg BFR mixture (*p* = 0.013 for males and *p* = 0.032 for females; Fig. 6A, B), however, these increases were not sustained at later ages. Serum levels of HDL cholesterol were elevated in both male and female offspring at PND 21 in the treatment groups exposed to either 20 mg/kg (*p* < 0.001 for both sexes) or 60 mg/kg (*p* = 0.002 and *p* < 0.001, for males and females, respectively; Fig. 6B, E). Levels of HDL remained elevated in males but not females at PND 46 (*p* = 0.003), but there were no differences in serum HDL observed in either sex at PND 210. In contrast, serum LDL cholesterol levels were reduced in pups of either sex at PND 21 in the treatment groups exposed to 20 or 60 mg/kg BFR mixture (*p* < 0.05 in males and *p* < 0.001 in females; Fig. 6C, F). In female offspring, LDL levels remained reduced in the 60 mg/kg group (*p* < 0.05) up to PND 210 (Fig. 6F). Furthermore, LDL levels were reduced in female offspring receiving 0.06 mg/kg BFR mixture at PNDs 46 and 210 when compared to controls (*p* < 0.05). Serum triglyceride levels were unaltered by BFR treatment in male and female offspring at all timepoints (data not shown).

Liver histology was investigated at PND 210 to assess evidence of metabolic syndrome‐related pathologies (i.e., hepatosteatosis). Of the specific traits examined (focal necrosis, portal leukocytic infiltrate, peribiliary inflammation, green pigment in Kupffer cells, bile duct hyperplasia, hepatocellular hypertrophy, hepatocellular vacuolization, and hepatocellular glycogen), there was no evidence of treatment‐induced pathology (Table 2). Pathologist scores did not exceed 2 in any animals (on a scale of 0–5, where 0 is minimal severity and 5 indicates a marked widespread lesion) and all damage was assessed to be mild or background.

**Table 2:** Effect of brominated flame retardant treatment on male and female offspring liver histology at postnatal day 210

	Dose, mg/kg							
	Male				Female			
	0	0.06	20	60	0	0.06	20	60
Number	5	5	5	5	5	5	5	5
Sinusoidal granuloma	0.80 ± 0.20	0.40 ± 0.25	0.40 ± 0.25	0.20 ± 0.20	0.60 ± 0.25	0.60 ± 0.25	0.40 ± 0.25	0.40 ± 0.25
Focal necrosis	0.80 ± 0.20	0.40 ± 0.25	1.0 ± 0	0.60 ± 0.25	1.0 ± 0	0.60 ± 0.25	0.60 ± 0.25	0.40 ± 0.25
Portal leukocytic infiltrate	0 ± 0	0.40 ± 0.25	0.40 ± 0.25	0.60 ± 0.25	0 ± 0	0.60 ± 0.25	0.60 ± 0.25	0 ± 0
Peribiliary inflammation	0 ± 0	0.20 ± 0.20	0 ± 0	0 ± 0	0 ± 0	0 ± 0	0 ± 0	0 ± 0
Green pigment in Kupffer cells	0 ± 0	0 ± 0	0 ± 0	0 ± 0	0 ± 0	0.40 ± 0.40	0 ± 0	0 ± 0
Bile duct hyperplasia	0 ± 0	0.20 ± 0.20	0 ± 0	0.20 ± 0.20	0 ± 0	0 ± 0	0 ± 0	0 ± 0
Hepatocellular hypertrophy	0 ± 0	0 ± 0	0 ± 0	0 ± 0	0 ± 0	0 ± 0	0 ± 0	0 ± 0
Hepatocellular vacuolation								
Random Cells	0 ± 0	0.40 ± 0.25	0 ± 0	0 ± 0	0 ± 0	0 ± 0	0 ± 0	0 ± 0
Zone 1	0 ± 0	0 ± 0	0 ± 0	0 ± 0	0 ± 0	0.40 ± 0.25	0.20 ± 0.20	0 ± 0
Hepatocellular glycogen								
Diffuse	0.40 ± 0.25	0 ± 0	0 ± 0	0.40 ± 0.40	0 ± 0	0 ± 0	0 ± 0	0 ± 0
Zones 1 and 2	0 ± 0	0.40 ± 0.40	0 ± 0	0 ± 0	0 ± 0	0 ± 0	0 ± 0	0 ± 0

Data expressed as mean score ± SEM.

## Discussion

In the current study, we describe the effects of perinatal exposure to an environmentally relevant BFR mixture on offspring outcomes with a focus on neurobehavioral and metabolic programming. We demonstrate that this exposure during in utero and postnatal life leads to mild neurobehavioral changes in Sprague–Dawley rats. We present novel findings regarding changes in serum cholesterol in the absence of alterations in glucose homeostasis or evidence of fatty liver, indicating that BFRs induce a transient change in cholesterol metabolism but have little impact on metabolic programming.

Although exposure to our BFR mixture influenced motor activity, as indicated by reduced vertical counts in pups of either sex at PND 21, the most sensitive indicator was an increase in emergence latency in exposed males, which was significantly delayed well after the cessation of treatment. Increased time for emergence into a novel, lighted area may indicate an increase in anxiety or fear of novelty. Our finding differs from previous studies of developmental neurotoxicity of brominated flame retardants that have investigated effects on anxiety‐related behaviors. Our previous study on the PBDE technical mixture DE‐71 using a similar experimental design, failed to observe any effects on anxiety‐related behaviors in offspring of either sex (Bowers et al., 2015). Similarly, no effects on performance in elevated T maze, an indicator of anxiety, were observed in adult mice exposed in utero until weaning to BDE‐47, a PBDE congener present in our mixture, although the same animals, when separated from their dam at a very young age, produced more intense vocalizations indicating heightened fear of separation (Ta et al., 2011). Others have reported results that could be interpreted as reduced anxiety in animals exposed to BFRs early in life. Adult male mice exposed early postnatally to BDE‐99 (also present in our mixture; Branchi et al., 2002) or BDE‐47 (Kuriyama et al., 2004) spent less time at the periphery of an open field than comparable unexposed mice. We did not observe any influence of our mixture on time spent at the periphery in our open field activity tests (data not shown). As our observations of increased time to emergence are not consistent with the available literature and are inconsistent with other observations in our study, it is not clear how to interpret these data.

It is not surprising that we observed a reduced frequency of rearing in PND 32 animals as altered rearing in open field activity is among the most common outcomes of neurodevelopmental toxicity of BFRs in rodents. Our previous study of the developmental neurotoxicity of technical mixture DE‐71, using a very similar study design, observed a dose‐dependent reduction in both locomotion and rearing in exposed pups at several ages, with females showing greater magnitude of effect (Bowers et al., 2015). A similar study in Wistar rats also showed an increase in the frequency of rearing at PND 60, but not other ages, in pups exposed in utero until weaning to repeated doses of DE‐71 (Kodavanti et al., 2010). Several studies (all from the same laboratory) have reported that early postnatal exposures of infant mice to a single dose of any of a variety of BDE congeners present in large amounts in our mixture (BDE‐99 [Eriksson et al., 2002]; BDE‐47 [Eriksson et al., 2001]; BDE‐153 [Viberg et al., 2003a]; and BDE‐209 [Viberg et al., 2003b]) caused a pattern of altered rates of locomotion and rearing over the course of the testing period in open field tests in adult animals. In each case, rearing frequency was reduced during early periods (0–20 min) of testing and increased rates later in testing (40–60 min). In addition, exposure of infant mice from PNDs 2 to 15 to BDE‐209 showed a reduction in locomotion and rearing when tested as adults (Rice et al., 2007). In contrast, early life exposures of mice BDE‐99 (from early pregnancy to weaning: [Branchi et al., 2002; Branchi et al., 2005]) or BDE‐47 (single gavage dose at PND 10: [Gee and Moser, 2008]) showed elevated rates of locomotion and rearing into adulthood. Similar results are also reported for rats exposed to BDE‐99 (Kuriyama et al., 2005). Our current results show no such change over time nor any persistence of this effect beyond PND 32. The sources of variability that could account for these inconsistencies in the reported effects of early life exposures to BFRs and neurobehavior in animal studies has been thoroughly reviewed (Costa and Giordano, 2007; Williams and DeSesso, 2010) and no obvious reason for discrepant results has been identified.

Several birth cohort studies have linked early exposures to BFR to later observations of fearful or anxiety‐like behaviors in humans. Behavioral assessment in formerly breast‐fed toddlers (about 36 months) observed that anxiety‐like behaviors were associated with higher levels of multiple PBDE congeners in maternal breast milk collected at 3 months postpartum (Adgent et al., 2014). A separate birth cohort study observed that gestational exposures to PBDEs was associated with reduced intelligence and increased hyperactivity, but not the anxiety‐related internalization scores, in children at 3 to 5 years of age (Chen et al., 2014).

Mixture exposure increased total and HDL cholesterol and reduced LDL cholesterol levels in serum of exposed offspring at PND 21 at the two highest doses. These differences from the control group diminished with age and there were no differences in any of these measures by PND 210. No effects on total serum cholesterol were observed in our previous BFR mixture studies with adult male (Ernest et al., 2012) or pregnant female rats (Berger et al., 2014), whereas increasing serum cholesterol has been reported from adult rats exposed for 28 days to doses of PBDEs much higher than levels in any diet in the current study (van der Ven et al., 2008; Oberg et al., 2010). Elevation in serum cholesterol and HDL in response to hypothyroidism is well documented in mammals (Dory and Roheim, 1981) and the elevation in pups in the current study are highly consistent with the ages and doses in which T4 was decreased by mixture treatment (Tung et al., 2016) suggesting that these are causally related. However, the treatment‐related reduction in serum LDL cholesterol is the opposite of what would be expected in response to hypothyroidism suggesting that serum LDL is influenced via some other unknown mechanism. Perinatal exposure to BFR was also observed to cause a minor reduction in the relative weight of various fat depots, but this only reached statistical significance in multiple depots in male pups at PND 46 and in periuterine fat pads in PND 210 females both in the 20 mg/kg and the (nominal) exposed group. Consistent with reduced fat depots, the body weights of male offspring exposed to either of the two highest doses were significantly reduced relative to control animals by treatment around PND 35 (Tung et al., 2016) and this was accompanied by a decrease in food consumption. The literature on developmental effects of PBDEs on fat accumulation are inconsistent with previous studies of perinatal DE‐71 exposure reported reductions (Kodavanti et al., 2010; Bowers et al., 2015), no change (Ellis–Hutchings et al., 2006; Bondy et al., 2013), mixed effects (Dunnick et al., 2012), or increases in pup body weights (Suvorov et al., 2009), although these studies did not measure any indicator of body fat content. Human cohort studies observed examining early life PBDE exposure are similarly inconsistent with PBDE exposures having no effect (Erkin–Cakmak et al., 2015) or leading to indications of reduced body fat in childhood (Hoffman–Riz et al., 2016; Vuong et al., 2016). These results do not support the idea that early life exposure to PBDEs or HBCDD contribute to increased weight gain later in life.

In addition to adiposity, hepatic lipid accumulation is associated with metabolic syndrome. Previous studies showed that hepatocellular vacuolization and lipid accumulation in the liver were increased in male offspring exposed perinatally to DE‐71 at 13 weeks of age (Dunnick et al., 2012). The current results do not support this finding, as histological assessment of liver sections at PND 210 did not reveal any notable changes in liver morphology, indicating that exposure to the BFR mixture does not induce liver steatosis.

Glucose homeostasis was also investigated in the current study. Contrary to previously published reports, exposure to this environmentally relevant mixture of BFRs did not alter fasting glucose and insulin serum levels (Suvorov et al., 2009; Nash et al., 2013). Adult male mice exposed to DE‐71 exhibited increased glucose‐to‐insulin ratios, suggesting that exposure to the technical mixture can alter glucose homeostasis (Dunnick et al., 2012; Nash et al., 2013). Similarly, human studies have revealed that serum PBDE levels are higher in people with diabetes (Lim et al., 2008; Zhang et al., 2016). However, in these studies, exposure occurred during adulthood with concurrent testing, whereas, in the current study, exposure occurred during early life with later testing. A separate study reported that perinatal exposure of Wistar rats to very low doses of BDE‐47 (a major congener of the DE‐71 that is present in the mixture tested in the current study) resulted in increased glucose uptake in adult male offspring by 75 days of age, suggesting that at least one component of our mixture may alter the developmental trajectory leading to altered glucose tolerance (Suvorov et al., 2009). Additionally, recent findings demonstrated that HBCDD‐exposed mice fed a high‐fat diet had liver lipid accumulation, elevated fasted glucose and insulin levels, and macrophage accumulation in adipose tissue (Yanagisawa et al., 2014). However, these effects were less prominent compared to a high‐fat diet or HBCDD treatment alone. Although our study did not challenge exposed animals with a high‐fat diet, our current results do not support the hypothesis that perinatal BFR exposure can predispose rats to develop obesity, glucose intolerance, or a fatty liver.

In conclusion, we present novel findings on the effects of exposure during the fetal and lactational windows of an environmentally relevant mixture of BFRs on neurodevelopment and metabolism. We observed a decrease in vertical movement and an increase in time to emerge to a lighted area in both male and female offspring; the latter occurred well after termination of exposure to the BFR mixture. A lack of observed effects on any of several measures of metabolic programming does not support the hypothesis that early exposure to BFR can predispose development of obesity or metabolic disease. These results contribute to our understanding of developmental toxicity of BFRs and lend further support to the conclusion that these may contribute to neurodevelopmental alterations.

## Supporting information

Supporting Information
