# Supplementary material for: Gestational and Lactational Exposure to an Environmentally‐Relevant Mixture of Brominated Flame Retardants: Effects on Neurodevelopment and Metabolism
Source: Birth Defects Res. 2017 Mar 24;109(7):497–512. doi: 10.1002/bdr2.1021 (PMC5434666; doi:10.1002/bdr2.1021)
Supplement: Supplementary file 1 — Supporting Information [file BDR2-109-497-s001.docx]

**Supplemental Table 1**: Glucose, Insulin and Homeostatic model assessment of insulin resistance for PND 25 rat pups exposed to the BFR-mixture.

|  |  | **0** | **0.06** | **20** | **60** |
| --- | --- | --- | --- | --- | --- |
| **Male** | N | 20 | 10 | 17 | 15 |
|  | glucose (mM) | 6.01 ± 0.15 | 6.20 ± 0.22 | 6.22 ± 0.12 | 6.34 ± 0.12 |
|  | insulin (pM) | 80.22 ± 3.70 | 108.53 ± 18.7 | 70.69 ± 4.62 | 75.07 ± 5.52 |
|  | HOMA-IR | 1.56 ± 0.07 | 2.10 ± 0.37 | 1.39 ± 0.08 | 1.7 ± 0.11 |
|  |  |  |  |  |  |
| **Female** | N | 20 | 11 | 16 | 16 |
|  | glucose (mM) | 6.12 ± 0.12 | 6.29 ± 0.16 | 5.99 ± 0.17 | 6.15 ± 0.15 |
|  | insulin (pM) | 101.94 ± 10.75 | 97.02 ± 15.15 | 75.05 ± 4.09 | 88.73 ± 7.31 |
|  | HOMA-IR | 1.98 ± 0.21 | 1.87 ± 0.29 | 1.46 ± 0.08 | 1.73 ± 0.14 |

**Supplemental Table 2**: Glucose, Insulin and Homeostatic model assessment of insulin resistance for PND 60 rat pups exposed to the BFR-mixture.

|  |  | **0** | **0.06** | **20** | **60** |
| --- | --- | --- | --- | --- | --- |
| **Male** | n | 20 | 10 | 17 | 15 |
|  | glucose (mM) | 6.20 ± 0.17 | 6.05 ± 0.26 | 5.89 ± 0.26 | 6.47 ± 0.30 |
|  | insulin (pM) | 128.56 ± 11.23 | 134.39 ± 18.34 | 120.26 ± 13.85 | 149.74 ± 18.27 |
|  | HOMA-IR | 2.47 ± 0.22 | 2.57 ± 0.35 | 2.30 ± 0.27 | 2.88 ± 0.34 |
|  |  |  |  |  |  |
| **Female** | n | 20 | 10 | 17 | 16 |
|  | glucose (mM) | 5.2 ± 0.16 | 5.53 ± 0.33 | 5.34 ± 0.19 | 5.16 ± 0.14 |
|  | insulin (pM) | 79.50 ± 4.79 | 102.26 ± 12.12 | 83.17 ± 7.13 | 80.06 ± 5.15 |
|  | HOMA-IR | 1.50 ± 0.09 | 1.88 ± 0.26 | 1.57 ± 0.14 | 1.50 ± 0.10 |

**Supplemental Table 3**: Glucose, Insulin and Homeostatic model assessment of insulin resistance for PND 90 rat pups exposed to the BFR-mixture.

|  |  | **0** | **0.06** | **20** | **60** |
| --- | --- | --- | --- | --- | --- |
| **Male** | n | 19 | 10 | 17 | 15 |
|  | glucose (mM) | 6.21 ± 0.20 | 6.23 ± 0.34 | 5.91 ± 0.16 | 5.94 ± 0.12 |
|  | insulin (pM) | 105.67 ± 7.31 | 128.44 ± 12.66 | 93.54 ± 8.06 | 99.85 ± 4.79 |
|  | HOMA-IR | 2.05 ± 0.15 | 2.49 ± 0.24 | 1.79 ± 0.15 | 1.93 ± 0.09 |
|  |  |  |  |  |  |
| **Female** | n | 18 | 10 | 17 | 16 |
|  | glucose (mM) | 5.14 ± 0.21 | 5.13 ± 0.23 | 5.21 ± 0.22 | 4.95 ± 0.16 |
|  | insulin (pM) | 68.44 ± 9.74 | 61.19 ± 6.62 | 62.85 ± 7.11 | 53.29 ± 5.57 |
|  | HOMA-IR | 1.28 ± 0.17 | 1.14 ± 0.13 | 1.18 ± 0.14 | 0.99 ± 0.11 |

**Supplemental Table 4**: Glucose, Insulin and Homeostatic model assessment of insulin resistance for PND 152 rat pups exposed to the BFR-mixture.

|  |  | **0** | **0.06** | **20** | **60** |
| --- | --- | --- | --- | --- | --- |
| **Male** | n | 19 | 10 | 17 | 15 |
|  | glucose (mM) | 6.18 ± 0.13 | 6.12 ± 0.24 | 6.18 ± 0.19 | 6.15 ± 0.15 |
|  | insulin (pM) | 107.25 ± 8.47 | 104.47 ± 14.18 | 106.03 ± 7.74 | 110.48 ± 10.99 |
|  | HOMA-IR | 2.08 ± 0.16 | 2.02 ± 0.27 | 2.05 ± 0.15 | 2.14 ± 0.21 |
|  |  |  |  |  |  |
| **Female** | n | 19 | 11 | 17 | 14 |
|  | glucose (mM) | 5.64 ± 0.17 | 5.6 ± 0.17 | 5.31 ± 0.12 | 5.46 ± 0.13 |
|  | insulin (pM) | 70.93 ± 10.76 | 60.74 ± 9.78 | 54.3 ± 4.74 | 63.13 ± 9.95 |
|  | HOMA-IR | 1.35 ± 0.19 | 1.20 ± 0.14 | 1.04 ± 0.09 | 1.20 ± 0.13 |

**Supplemental Table 5**: Glucose, Insulin and Homeostatic model assessment of insulin resistance for PND 180 rat pups exposed to the BFR-mixture.

|  |  | **0** | **0.06** | **20** | **60** |
| --- | --- | --- | --- | --- | --- |
| **Male** | n | 18 | 10 | 17 | 15 |
|  | glucose (mM) | 5.41 ± 0.12 | 5.54 ± 0.22 | 5.45 ± 0.16 | 5.20 ± 0.15 |
|  | insulin (pM) | 108.32 ± 11.17 | 79.01 ± 15.55 | 101.58 ± 19.02 | 94.34 ± 17.74 |
|  | HOMA-IR | 1.96 ± 0.21 | 1.53 ± 0.29 | 1.89 ± 0.33 | 1.73 ± 0.35 |
|  |  |  |  |  |  |
| **Female** | n | 20 | 11 | 17 | 15 |
|  | glucose (mM) | 4.71 ± 0.14 | 5.26 ± 0.27 | 4.75 ± 0.15 | 4.71 ± 0.12 |
|  | insulin (pM) | 57.87 ± 7.65 | 75.79 ± 20.32 | 52.57 ± 10.56 | 76.97 ± 16.29 |
|  | HOMA-IR | 1.09 ± 0.14 | 1.41 ± 0.36 | 0.99 ± 0.18 | 1.78 ± 0.43 |
